# A Nerve Growth Factor Dipeptide Mimetic Stimulates Neurogenesis and Synaptogenesis in the Hippocampus and Striatum of Adult Rats with Focal Cerebral Ischemia

**DOI:** 10.32607/20758251-2019-11-3-31-37

**Published:** 2019

**Authors:** T. A. Gudasheva, P. Yu. Povarnina, A. A. Volkova, S. V. Kruglov, T. A. Antipova, S. B. Seredenin

**Affiliations:** Federal State Budgetary Institution “Zakusov Research Institute of Pharmacology”, Baltiyskay Str. 8 , Moscow, 125315, Russia

**Keywords:** NGF, GK-2 dipeptide mimetic, stroke, neurogenesis, synaptogenesis, Ki67, PSD-95, synaptophysin

## Abstract

The nerve growth factor (NGF) and its mimetics, which have neuroprotective and neuroregenerative properties, are attractive candidates for developing new drugs for brain injury therapy. A dipeptide mimetic of NGF loop 4, bis(N-succinyl-L-glutamyl-L-lysine) hexamethylenediamide (GK-2), developed at the Zakusov Research Institute of Pharmacology, has the NGF-like ability to activate TrkA receptors, but unlike NGF, GK-2 activates mainly the PI3K/AKT pathway associated with neuroprotection and has no effect on the MAPK cascade associated with hyperalgesia, the main side effect of NGF. That GK-2 possesses neuroprotective activity has been observed in various models of cerebral ischemia. GK-2 was found to statistically significantly reduce the cerebral infarct volume in experimental stroke, even at treatment onset 24 h after injury. This suggests that GK-2 possesses neuroregenerative properties, which may be associated with the activation of neurogenesis and/or synaptogenesis. We studied the effect of GK-2 on neurogenesis and synaptogenesis in experimental ischemic stroke caused by transient occlusion of the middle cerebral artery in rats. GK-2 was administered 6 or 24 h after surgery and then once a day for 7 days. One day after the last administration, proliferative activity in the hippocampus and striatum of the affected hemisphere was assessed using Ki67 and synaptogenesis in the striatum was evaluated using synaptophysin and PSD-95. Ki67 immunoreactivity, both in the striatum and in the hippocampus of the ischemic rats, was found to have dropped by approximately 30% compared to that in the sham-operated controls. Synaptic markers - synaptophysin and PSD-95 - were also statistically significantly reduced, by 14 and 29%, respectively. GK-2 in both administration schedules completely restored the level of Ki67 immunoreactivity in the hippocampus and promoted its increase in the striatum. In addition, GK-2 restored the level of the postsynaptic marker PSD-95, with the therapeutic effect amounting to 70% at the start of its administration after 6 h, and promoted restoration of the level of this marker at the start of administration 24 h after an experimental stroke. GK-2 had no effect on the synaptophysin level. These findings suggest that the neurotrophin mimetic GK-2, which mainly activates one of the main Trk receptor signaling pathways PI3K/ AKT, has a stimulating effect on neurogenesis (and, probably, gliogenesis) and synaptogenesis in experimental cerebral ischemia. This effect may explain the protective effect observed at the start of dipeptide administration 24 h after stroke simulation.

## INTRODUCTION

The development of pathogenic treatments for an ischemic stroke after reperfusion remains a challenge in modern medicine.

The nerve growth factor (NGF) is an attractive neuroprotective agent capable of affecting the main mechanisms of ischemic neuronal injury: glutamate toxicity mediating excessive calcium entry into the cell [1]; oxidative stress [2]. NGF reduces the expression of pro-apoptotic proteins and activates the synthesis of anti-apoptotic proteins [3, 4]. NGF was experimentally proven to be involved in neurogenesis in the adult brains of rodents. NGF enhances the proliferation of neuronal stem cells, promotes the survival of progenitor cells, and stimulates the differentiation of neuroblasts in both major neurogenic zones: the subventricular zone and the dentate gyrus of the hippocampus [5-9]. The efficacy of intracerebral and intranasal administration of NGF has been proven in various cerebral ischemia models in rodents. It is extremely important that the neurotrophin remains active upon delayed administration – 24 h after a simulated stroke [6, 10]. Applications of NGF in clinical practice are limited not only due to its unsatisfactory pharmacokinetic properties, but also due to serious pleiotropic side effects, such as hyperalgesia, catastrophic weight loss, excessive neuritogenesis, and angiogenesis [11]. Probably, the disadvantages of the full-length NGF protein may be obviated through the development of related low-molecular-weight mimetics with a selective pharmacological activity [12].

At the Zakusov Research Institute of Pharmacology, a hypothesis has been developed, holding that receptor recognition and binding are controlled by the most exposed portions of neurotrophin loops, usually by the central parts of their beta-turns, with mimetics of beta-turns from different loops imitating different functions of the neurotrophin [13]. This hypothesis was the basis for the development of a dimeric dipeptide mimetic of NGF loop 4, bis(N-monosuccinyl-L-glutamyl- L-lysine) hexamethylenediamide, that received the laboratory code GK-2 [RF Patent No. 2410392, 2010; US patent US 9,683,014, 2017; Chinese Patent CN 102365294 B, 2016]. GK-2 was found to have the NGF-like ability to activate TrkA receptors, but unlike the full-length protein, GK-2 primarily activates the PI3K/AKT pathway associated with neuroprotection and has no effect on the MAPK cascade that mediates hyperalgesia [14].

In vitro studies have shown that GK-2 in micronanomolar amounts, like NGF, has a neuroprotective effect on both immortalized and primary neuron cultures under oxidative stress and glutamate toxicity [15]. The neuroprotective activity of GK-2 was also demonstrated in vivo upon systemic administration in Alzheimer and Parkinson models, as well as in various cerebral ischemia models [16]. In support of the in vitro data, GK-2, unlike the full-length protein, did not cause hyperalgesia and weight loss in in vivo experiments [14]. A study of the neuroprotective effect of GK-2 in a model of ischemic stroke caused by transient occlusion of the middle cerebral artery in rats showed that the dipeptide statistically significantly reduced the amount of brain injury if the onset of therapeutic administration started between 4 and 24 h, with the strongest effect (60%) being observed if the first administration occured 6 h after surgery [17]. Preservation of the GK-2 activity when its first administration occurs 24 h after a simulated ischemic stroke is probably not explained by its neuroprotective properties, because the infarction area has fully formed by that time [18]. Therefore, the protective effect of GK-2 administered 24 h after an ischemic stroke may be associated with the regenerative properties of the mimetic.

To shed light on this issue, we studied the effect of GK-2 on neurogenesis and synaptogenesis in an experimental ischemic stroke using antibodies to the Ki67 proliferation marker and synaptogenesis markers (synaptophysin and PSD-95). Neurogenesis and synaptogenesis parameters were assessed in the hippocampus and striatum. These structures were chosen because one of the main neurogenic zones in the adult brain (subgranular zone) is located in the hippocampus, and the striatum is the most affected structure during occlusion of the middle cerebral artery [6]. The effects of GK-2 were studied when its administration occurred 6 and 24 h after a simulated stroke.

## EXPERIMENTAL

**Animals**

We used 34 male Wistar rats weighing between 220 and 250 g and 8–9 weeks of age at the beginning of the experiment. The animals originated from the Andreevka Branch of the Scientific Center of Biomedical Technologies of the FMBA, Russia. The animals were kept in a vivarium under natural circadian light/dark cycles with free access to standard granular feed and water. The study complied with the requirements of Order of the Ministry of Health of the Russian Federation No. 199 “On Approval of the Rules of Good Laboratory Practice” and Decision of the Council of the Eurasian Economic Commission No. 81 “On Approval of the Rules of Good Laboratory Practice of the Eurasian Economic Union in the Area of Circulation of Medicines.” All manipulations with the animals were approved by the Bioethical Commission of the Zakusov Research Institute of Pharmacology.

**Simulation of ischemic stroke**

Ischemic stroke was simulated by intravascular thread occlusion of the middle cerebral artery [19]. All surgical procedures were performed using titanium microsurgical tools. The rats were anesthetized with a 5% chloral hydrate solution (350 mg/kg, ip). Following a midline incision in the neck, the right carotid triangle was identified, which is bounded superiorly by the digastric muscle, laterally by the sternocleidomastoid muscle, and medially by the sternohyoid muscle. In the carotid triangle, the carotid neurovascular bundle formed by the common carotid artery and the vagus nerve was identified. The vagus nerve was carefully isolated, and a microsurgical vascular titanium clip was placed on the common carotid artery 1.5 cm below its bifurcation. The external and internal carotid arteries were carefully isolated from adhesions. The external carotid artery was tightly tied with a cotton suture. The internal carotid artery was loosely tied with a vicryl suture, and then the external carotid artery was cut proximal to the suture. A 0.25-mm heparinized nylon filament was inserted through the stump of the external carotid artery into the internal carotid artery to a depth of 19–21 mm (until the middle cerebral artery was occluded) and fixed with a microvascular clip. The circulation was occluded for 60 min; then, the filament was removed from the vessel, restoring blood supply in the middle cerebral artery territory. After filament removal, the stump of the external carotid artery was tightly sealed by electrocautery coagulation. Sham-operated animals underwent the same procedures, except for vascular transection and filament insertion. The midline incision in the neck was sutured with a cotton thread and treated with streptocide. In our experiments, the ischemic injury volume on day 7 after the simulated stroke was 700–800 mm3 (according to the morphometry of brain sections stained with 2,3,5-triphenyltetrazolium chloride) [17].

**Study design**

**Fig. 1:**
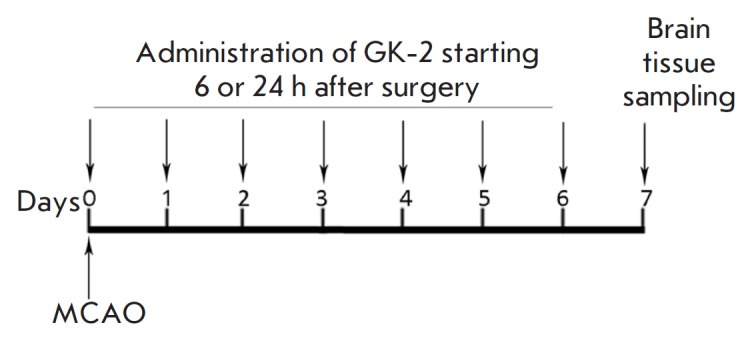
Experimental design. MCAO – middle cerebral artery occlusion

Operated animals were randomly divided into three groups: the “stroke” group (operated untreated animals; n = 9) and two groups receiving GK-2 (1 mg/kg, ip) diluted in water for injection. GK-2 was administered in a volume of 2 mL per kilogram of body weight, starting 6 (n = 10) or 24 h (n = 7) after surgery, and then once a day, with the end of administration on day 6 after surgery. Animals from the groups “stroke” and “sham surgery” (n = 8) received water for injection according to the same schedule. On day 7 after surgery, the rats were decapitated; the brain was removed at a temperature of 0–4°C; and the striatum and hippocampus were removed from the affected hemisphere, frozen in liquid nitrogen, and stored at –70°C. The experimental design is shown in Fig. 1.

**Assessment of the proliferative activity and synaptogenesis**

The effect of GK-2 on the proliferative activity and synaptogenesis in the striatum and hippocampus was evaluated using a Western blot analysis [20]. After thawing, animal tissue samples from one group were combined to prepare at least three samples. Then, the samples were homogenized at 4°C in a glass homogenizer with lysis buffer (50 mM Tris-HCl, 5 mM EDTA, 1 mM dithiothreitol, 1% Triton X-100, pH 7.5) containing a cocktail of protease inhibitors (pepstatin, bestatin, leupeptin, and aprotinin; Sigma-Aldrich, USA), at a tissue : buffer ratio of 1 : 10 (weight/volume). Then, the samples were incubated at 4°C for 20 min and centrifuged (15,000 rpm for 20 min; Allegra® X-12R centrifuge; BeckmanCoulter Inc., USA) at the same temperature. The protein concentration in the supernatant was determined according to the Folin–Lowry method [21]. Supernatant proteins were separated on a 12% polyacrylamide gel and transferred onto a polyvinylidene fluoride membrane by electroelution. The membranes were then incubated in Tris-HCl buffer (200 mM, pH 7.5) containing 1% Tween-20 (TBST) and 5% (w/v) skim milk at room temperature for 1 h. Then, the membranes were incubated with primary monoclonal antibodies against synaptophysin (BD Biosciences, Great Britain) at a 1 : 5,000 dilution, primary monoclonal antibodies against PSD-95 (Thermo Fisher Scientific, USA) at a 1 : 1,000 dilution, and primary polyclonal antibodies against Ki67 (Thermo Fisher Scientific) at a 1 : 5,000 dilution at room temperature for 1.5 h; excess antibodies were removed with TBST containing 0.5% (w/v) skim milk. The membranes were incubated with secondary goat antibodies against rabbit IgG (Santa Cruz Biotechnology, USA; 1 : 2,000) conjugated to horseradish peroxidase at room temperature for 1 h. After the removal of secondary antibodies with TBST, the proteins were detected by a reaction with enhanced chemiluminescence reagents (ECL reagents, Santa Cruz Biotechnology) using the Alliance Q9 gel documentation system (UVITEC, UK). Images were analyzed using the GIMP2 software.

**Statistical processing**

Intergroup differences were evaluated using a Mann– Whitney U test. Differences were considered statistically significant at p < 0.05. Data was presented as a mean and a standard error of the mean.

## RESULTS AND DISCUSSION

**Fig. 2:**
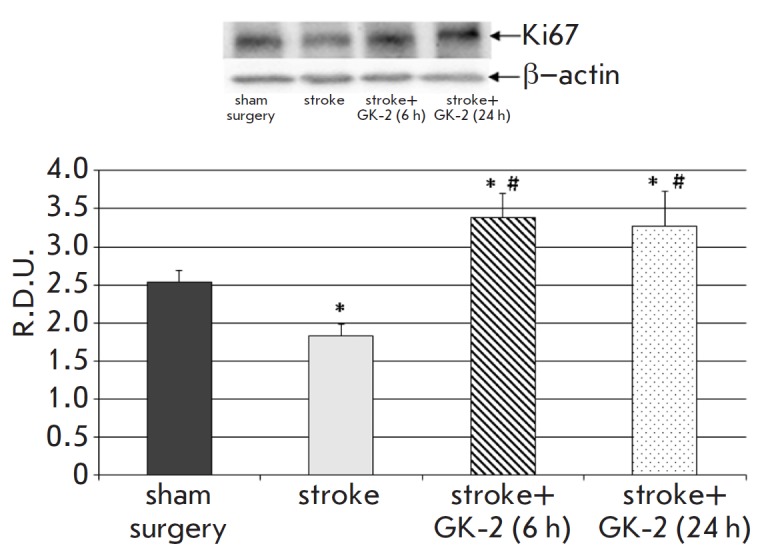
Effect of GK-2 on Ki67 proliferation marker levels in the hippocampus upon subchronic (7 days) administration (1 mg/kg, ip) after an experimental ischemic stroke caused by transient occlusion of the middle cerebral artery in rats, in a 6 or 24 h therapeutic window (the time between surgery and the first injection of the agent). R.D.U. – relative densitometric units. * – p < 0.05 compared to the sham-operated group, # – p < 0.05 compared to the stroke group (Mann–Whitney U test)

**Fig. 3:**
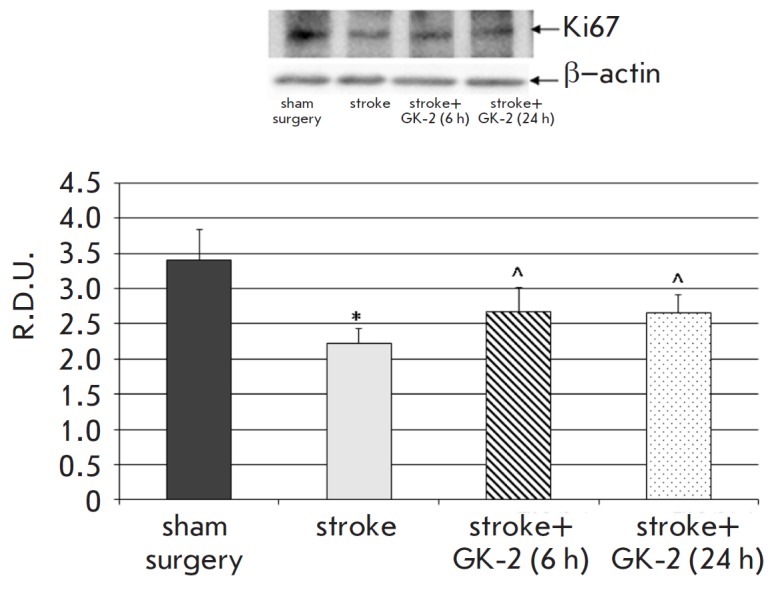
Effect of GK-2 on Ki67 proliferation marker levels in the striatum upon subchronic (7 days) administration (1 mg/kg, ip) after an experimental ischemic stroke caused by transient occlusion of the middle cerebral artery in rats, in a 6 or 24 h therapeutic window (the time between surgery and the first injection of the agent). R.D.U. – relative densitometric units. * – p < 0.05 compared to the sham-operated group; ^ – p < 0.1 compared to the stroke group (Mann-Whitney U test)

Seven days after surgery, the immunoreactivity for the Ki67 proliferation marker, both in the striatum and in the hippocampus of the ischemic brain of rats untreated with GK-2, was reduced by about 30% compared to that in the sham-operated controls (Fig. 2, Fig. 3). Administration of GK-2 to ischemic animals for seven days led not only to a restoration of the Ki67 immunoreactivity level in the hippocampus, but also to an excess of its baseline values by 35 and 36% for a 6- and 24-hour interval between surgery and the first peptide administration, respectively (Fig. 2). These findings indicate the ability of the NGF mimetic to stimulate a proliferative activity in the hippocampus of the ischemic brain. Based on previously obtained data on the improvement in the neurological status of rats receiving GK-2 (administration starting 6 and 24 h after surgery) under the same conditions as in this experiment [17], we suggest that dipeptide-induced stimulation of proliferative activity leads, at least predominantly, to neurogenesis. This is also supported by published data indicating that the full-length NGF stimulates hippocampal neurogenesis, increasing the proliferative activity and promoting the survival of neuroblasts in the dentate gyrus of the hippocampus [5, 9].

Therefore, we may draw a preliminary conclusion that GK-2 reproduces the effects of NGF, increasing hippocampal neurogenesis in cerebral ischemia. An effect of GK-2 on gliogenesis is less likely, because even full-length neurotrophin primarily stimulates neuroblast formation.

In the striatum, GK-2 administered in both schedules increased the level of Ki67 immunoreactivity about 1.2-fold compared to that in the untreated animals (which corresponds to a therapeutic effect of 36–37% (p = 0.08)) (Fig. 3). During middle cerebral artery occlusion, NGF is known to stimulate the proliferative activity and increase the survival of neuroblasts in the subventricular zone and striatum of rats [6, 10]. In the same brain ischemia model, by using the BrdU proliferation marker, intranasal administration of NGF was shown to increase the survival rate of progenitor cells in the striatum about 1.5-fold after 4 weeks [6]. By using another proliferation marker, Ki67, NGF expressed in the rat brain using a lentiviral vector was found [10] to stimulate neurogenesis in the injured striatum, increasing the number of neuroblasts about 2-fold compared to that in untreated animals 3 weeks after a simulated stroke.

Therefore, systemically administered GK-2 apparently has an effect on neurogenesis in the striatum of ischemic rats, which is similar to that of NGF introduced in the brain intranasally or by gene therapy.

To date, a large amount of experimental data has been accumulated indicating a compensatory role for neurogenesis in the subventricular and subgranular zones in cerebral ischemia [22]. According to the published data, neurogenesis is activated in pathological conditions; in this case, newly formed neuroblasts migrate to injured brain areas, where they replace dead neurons [23]. Therefore, it may be assumed that the stimulating effect of GK-2 on the proliferative activity in the hippocampus leads to the activation of neuroregenerative processes in the ischemic injury area due to the migration of a larger amount of survived neuronal progenitor cells into this area and, ultimately, to their integration. Probably, the GK-2-induced increase in the Ki67 proliferation marker level in the striatum is related to the migration of neuroblasts from neurogenic zones to this region.

**Fig. 4:**
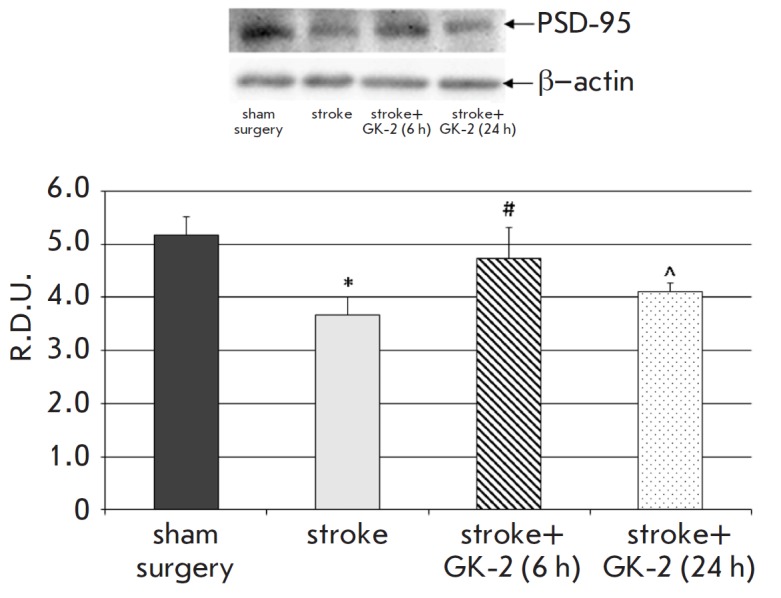
Effect of GK-2 on PSD-95 levels in the striatum upon subchronic (7 days) administration (1 mg/kg, ip) after an experimental ischemic stroke caused by transient occlusion of the middle cerebral artery in rats. Administration of GK-2 was begun 6 or 24 h after surgery. R.D.U. – relative densitometric units. * – p < 0.05 compared to the sham-operated group; # – p < 0.05 compared to the stroke group; ^ – p < 0.1 compared to the stroke group (Mann-Whitney U test)

**Fig. 5:**
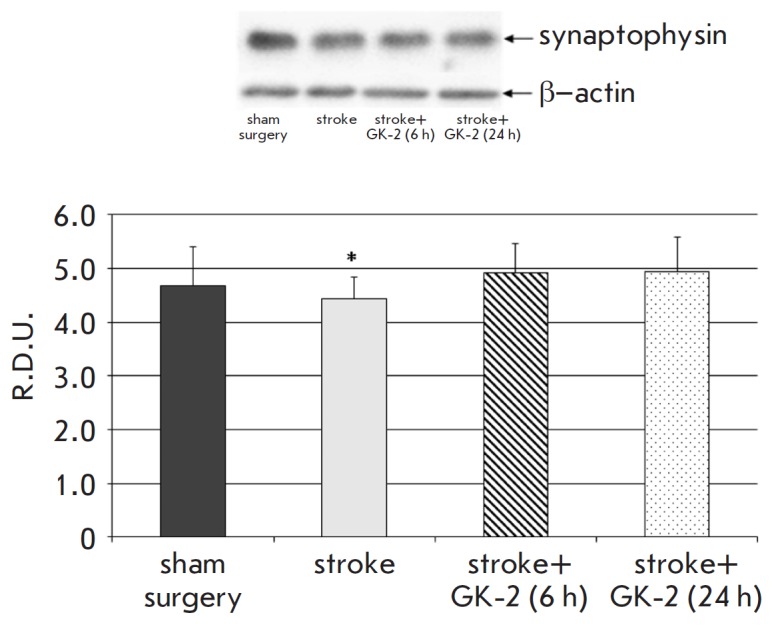
Effect of GK-2 on synaptophisin presynaptic marker levels in the striatum upon subchronic (7 days) administration (1 mg/kg, ip) after an experimental ischemic stroke caused by transient occlusion of the middle cerebral artery in rats. Administration of GK-2 was started 6 or 24 h after surgery. R.D.U. – relative densitometric units. * – p < 0.05 compared to the sham-operated group (Mann-Whitney U test)

Assessment of the synaptic marker levels in the striatum of the injured hemisphere in ischemic rats showed that the levels of both the PSD-95 postsynaptic density protein and the presynaptic synaptophysin protein were statistically significantly lower compared to those in sham-operated animals (29 and 14%, respectively) (Fig. 4, Fig. 5). These results confirm the presence of an ischemic injury in this area, which is associated with a loss of neurons and synapses. GK-2 restored the PSD-95 level with a therapeutic effect of 70% when its administration started 6 h after surgery; however, administration of GK-2 starting 24 h after surgery caused only a tendency (p = 0.08) to restore the level of this marker (Fig. 4).

At the same time, GK-2 had no statistically significant effect on the synaptophysin level in the striatum (Fig. 5).

A possible explanation for the lack of changes in the presynaptic synaptophysin protein upon administration of GK-2 is the formation of presynaptic terminals at the final stages of neurogenesis, which completes in at least 3 weeks. On the other hand, the postsynaptic density protein PSD-95 is a component of the dendritic spines that form in a shorter time [24]. On the basis of these findings, we may suggest that GK-2 stimulated striatal synaptogenesis in terms of dendritic spine formation 7 days after an experimental stroke.

**Table 1:** Neuroprotective and neuroregenerative effects of GK-2 (1 mg/kg, 7 days) in a model of ischemic stroke caused by transient occlusion of the middle cerebral artery in rats

First administration after surgery, h	Reduction in the ischemic injury volume [17], %	Stimulation of neurogenesis (based on the Ki67 proliferation marker), therapeutic effect,%		Stimulation of synaptogenesis in the striatum (based on the postsynaptic marker PSD-95), therapeutic effect, %
		hippocampus	striatum	
6	60^*^	220^*^	37^^^	72^*^
24	24^*^	205^*^	36^^^	30^^^

*Note:*The therapeutic effect was calculated using the formula: [(protein level in the stroke + GK-2 group – protein level in the stroke group)/(protein level in the sham-surgery group – protein level in the stroke group)] × 100% * – p < 0.05; ^ – p < 0.1 compared to the stroke group (Mann–Whitney U-test).

To assess the contribution of neuroregeneration to the protective effects of GK-2 in an experimental stroke, we compared our biochemical data with the results of a previous morphological study [17] on the role of GK-2 in reducing the volume of an ischemic brain injury. All dipeptide effects were evaluated on the 7^th^ day after surgery (see Table).

As seen from Table, GK-2 reduced the volume of the ischemic injury by 60% and 24% when administration started 6 and 24 h after surgery, respectively. According to [18], a significant amount of penumbra neurons are preserved 6 h after a stroke, which may survive thanks to the neuroprotective effect of GK-2. Probably, recovery in the brain occurs partially due to neurogenesis. In the absence of a penumbra, restoration of injured brain tissue (by 24%) after 24 h may occur only due to regenerative processes. These findings suggest that regeneration associated with the proliferation and migration of new cells does not depend on the penumbra volume, which is evidenced by similar indicators of Ki67 immunoreactivity upon GK-2 administration starting 6 and 24 h after surgery.

However, given the PSD-95 postsynaptic marker levels, synaptogenesis may depend on the total amount of intact neurons, both survived and newly formed. Changes in the density of this marker in the presence of GK-2 are proportional to the degree of neuronal volume recovery in the ischemic injury area (72/60 and 30/24, respectively).

Therefore, our findings suggest that the effect of GK-2 administered shortly after a simulated stroke is associated with both neuroprotective and reparative processes; while the effect of GK-2 administered after 24 h is associated with the stimulation of reparative processes, including both neurogenesis (and, probably, gliogenesis) and synaptogenesis.

## CONCLUSION

The dimeric dipeptide mimetic of nerve growth factor loop 4, GK-2, administered subchronically and starting 6 and 24 h after surgery statistically significantly restores the reduced proliferation of neuronal stem cells in the hippocampus and increases the proliferative activity in the striatum, according to the Ki67 marker, in an experimental ischemic stroke caused by transient occlusion of the middle cerebral artery. Based on the previously obtained data on the improvement in the neurological status of rats receiving GK-2 under conditions similar to those of the present experiment [17], we may suggest that stimulation of proliferative activity by the dipeptide leads, at least primarily, to neurogenesis. The effect of GK-2 increases the number of synaptic contacts being reduced after surgery, which was assessed using the postsynaptic marker PSD-95, upon administration starting 6 h after surgery and increases this indicator when the first administration occurrs 24 h after surgery. These findings demonstrate the stimulating effect of GK-2 on neurogenesis (and, probably, gliogenesis) and synaptogenesis in experimental cerebral ischemia.
